# microRNAs as novel biomarkers of schizophrenia (Review)

**DOI:** 10.3892/etm.2014.2014

**Published:** 2014-10-09

**Authors:** JIE WANG, YUHAN WANG, JIANBO YANG, YUANSHUAI HUANG

**Affiliations:** 1 Department of Laboratory Medicine, Luzhou Medical College, Luzhou, Sichuan 646000, P.R. China; 2 Department of Nuclear Medicine, Affiliated Hospital of Luzhou Medical College, Luzhou, Sichuan 646000, P.R. China; 3 Department of Blood Transfusion, Affiliated Hospital of Luzhou Medical College, Luzhou, Sichuan 646000, P.R. China

**Keywords:** schizophrenia, microRNA, etiology, diagnosis, biomarker

## Abstract

Schizophrenia is a severe mental illness and the most common complex neuropsychiatric disorder. To date, the etiology of schizophrenia is unclear; consequently, the diagnosis of schizophrenia is controversial. Biomarkers that reflect the dysregulations observed in schizophrenia may potentially assist the diagnosis of the disorder. However, the majority of these biomarkers are found in the brain tissue, which is not readily accessible, leading to the search for novel biomarkers that are present in the peripheral blood. microRNAs (miRNAs) are small non-coding RNAs that function as post-transcriptional regulators of gene expression. Previous investigations have revealed that miRNAs may play a potential role in neurodevelopment and maturation, including neurite outgrowth, dendritogenesis and dendritic spine formation. These developments highlight the significance of miRNAs as potential diagnostic biomarkers for schizophrenia. To date, miRNA biomarkers have been predominantly extracted from the brain tissue; however, future studies should examine the use of peripheral blood miRNAs as biomarkers, as these are more accessible.

## 1. Introduction

### Schizophrenia is a common mental disorder

Schizophrenia is a severe psychiatric disorder that has been found to have a lifetime prevalence of ~1% in a number of population studies (1–3). The condition is characterized by impaired cognition, positive psychotic symptoms, including hallucinations, delusions and disorganized behavior, and negative symptoms, such as social withdrawal and apathy (2). Schizophrenia is a ‘complex genetic disease’, with an etiology involving multiple genetic and environmental factors. The genetic contribution is significant, since schizophrenia has been shown to have a heritability risk of ~80%, with the risk decreasing by ~50% for each degree of family relation (3).

### microRNAs (miRNA) are the unseen regulators of gene expression

miRNAs are a class of small RNAs found only in eukaryotes, which were first identified less than two decades ago. Modulation of gene expression does not rely solely on regulatory proteins. miRNAs are single-stranded RNA molecules, consisting of 21–23 nucleotides, that are not translated into proteins, but are key to the complex cell machinery responsible for gene expression (4). A previous study estimated that one third of human genes are directly targeted by miRNAs (5). In an attempt to clone the gene responsible for the lin-4 phenotype in roundworms, Lee *et al* identified miRNAs for the first time as small non-coding RNA molecules (6). To date, 2,652 mature human miRNAs have been identified (www.mirbase.org; accessed 20, June 2013).

**Figure 1:**
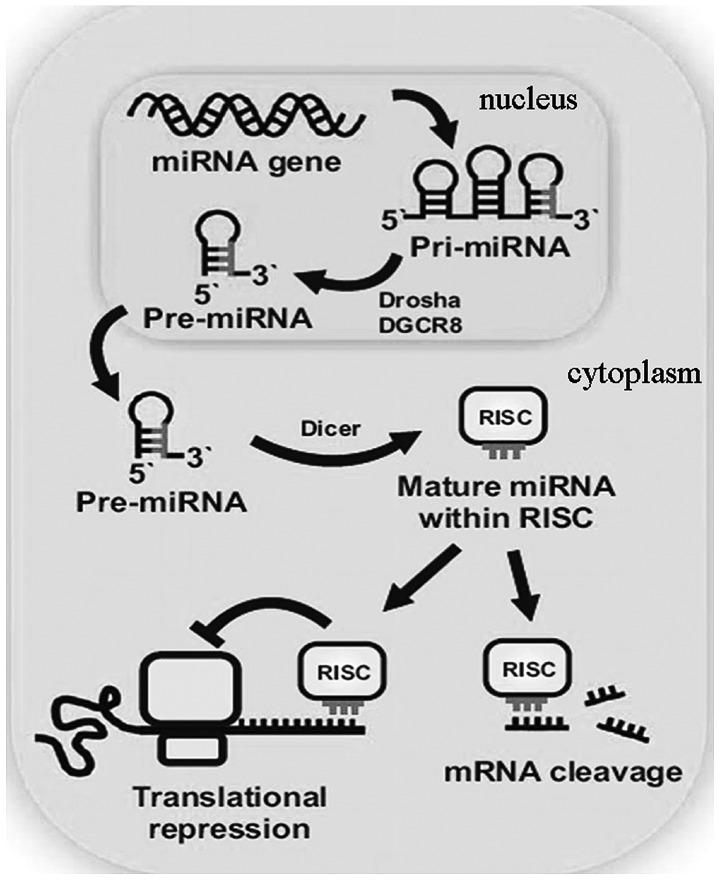
Biogenesis of miRNA [adapted from Bravo and Dinan, 2011 (3)]. Mature miRNA is much smaller compared with the gene involved in encoding the molecule. Pri-miRNA has a hairpin structure with a poly(A) tail and cap. The hairpin molecule is processed by the nuclease, Drosha, and the RNA-binding protein, DGCR8 (known as Pasha in invertebrates), and the resulting molecule is known as pre-miRNA. The small pre-miRNA passes from the nucleus into the cytosol and is processed by the endonuclease, Dicer. Mature miRNA forms part of the RISC and the complex provides the gene silencing capacity of miRNA. miRNA, microRNA; pri-miRNA, primary-miRNA; pre-miRNA, precursor-miRNA; RISC, RNA-induced silencing complex.

The biogenesis of miRNAs is initiated by transcription from intergenic or intron genomic regions into primary-miRNA molecules (pri-miRNAs). Pri-miRNAs are cleaved inside the nucleus by the components of the microprocessor complex, consisting of Drosha and DGCR8, to generate RNA hairpins, known as precursor-miRNA molecules (pre-miRNAs). Pre-miRNA is exported into the cytoplasm and is further cleaved by the RNaseIII enzyme, Dicer; therefore, a duplex of two miRNA strands is formed. Next, the miRNA duplex is unwound and one of the strands is incorporated into a large miRNA-induced silencing complex, which participates in the detection and binding to the 3′-untranslated region of messenger RNAs (mRNAs). As a result, translation is inhibited. However, the underlying mechanisms are yet to be fully understood (Fig. 1) (7).

### Genetic basis of miRNA abnormalities in schizophrenia

A hemizygous deletion of a 1.5–3-Mb region of chromosome 22 can lead to the 22q11 deletion syndrome (22q11DS), which is characterized by multiple physical and psychiatric abnormalities. A previous study determined that ~30% of 22q11DS patients may develop schizophrenia (8). miR-25 and miR-185 are regulators of the sarco/endoplasmic reticulum Ca^2+^ ATPase (SERCA2), which is responsible for loading Ca^2+^ into the endoplasmic reticulum. Earls *et al* found that miR-25 and miR-185 were depleted in mouse models of 22q11DS and restoration of these miRNAs to presynaptic neurons rescued the long-term potentiation of DGCR8^+/−^ mice (1). The authors concluded that miRNA-dependent SERCA2 dysregulation is a pathogenic event in 22q11DS and schizophrenia.

## 2. miRNAs and schizophrenia

### miRNA function in the nervous system

The intricate architecture of the nervous system and the ability of the neurons for postsynaptic remodeling requires the coordination of complex intracellular networks consisting of molecular signal transduction systems. Due to the abundance of neural networks, gene variants are able to cause system dysfunctions, manifesting as associated neurobehavioral syndromes. Previous studies revealed that post-transcriptional gene regulation by miRNA is an important factor shaping the topography of the neural networks. Over half of miRNAs identified have been shown to be highly or exclusively expressed in the brain, a number of which have been implicated in important aspects of neuronal function (9). miR-124 and miR-9 play a crucial role in neurogenesis; overexpression of these miRNAs decreases the number of astrocytes, whereas inhibition of these miRNAs reduces the number of neurons (10). miR-134 was found to regulate the size of dendritic spines and enrich the synapse dendritic region of rat hippocampal neurons (11). In addition, miR-134 regulates the translation of Limk1, which is a protein kinase that affects dendritic spine morphology via the regulation of actin filaments (5).

### Dysregulation of miRNA in schizophrenia

miRNA has received increasing attention in genetic studies of schizophrenia. A number of studies support the hypothesis that miRNA plays an important role not only in human brain development, but also in brain diseases (12–16). Hunsberger *et al* hypothesized that miRNA may serve as a unifying link among the structural developmental anomalies, neurotransmitter alterations and response to treatment in schizophrenia (17). The schizophrenia-associated miRNAs are summarized in Table I.

Alterations in the sequence of certain miRNAs leads to the alteration of gene regulation, which contributes to the development of a psychiatric disorder. Perkins *et al* compared the expression of 264 miRNAs from the prefrontal cortex of patients diagnosed with schizophrenia and 21 individuals not suffering from a psychiatric illness, used as controls (18). Using a custom-made miRNA microarray, the authors identified that the expression of 15 miRNAs decreased and the expression of one miRNA increased in the prefrontal cortex of the schizophrenia patients, when compared with the control individuals.

In addition, Guo *et al* demonstrated that miR-195 is involved in a complex regulatory network, which affects the signaling pathways considered to be significant in the development of schizophrenia (19).

The gene encoding miR-346 is located in the intron of the glutamate receptor ionotropic δ1 (GRID1) gene, which is known to be involved in schizophrenia susceptibility (5). Using quantitative polymerase chain reaction, Zhu *et al* detected the expression levels of miR-346 and GRID1 in brain RNA samples of 35 patients with schizophrenia and 34 controls, obtained from the Stanley Medical Research Institute (Chevy Chase, MD, USA) (3). The expression levels of miR-346 and GRID1 were found to be lower in the schizophrenia patients compared with the controls.

In a study of the Chinese population, Xu *et al* described a potentially functional variant that affected pre-miR-30e and was closely associated with schizophrenia (28). The variant affected the predicted structure and the release of pre-miRNA, as well as the accuracy of the mature miRNA. Despite the samples being obtained from the peripheral blood, the findings of Xu *et al* were comparable to the observations of Perkins *et al* (18), who detected an increase in the expression level of miR-30e in the prefrontal cortex of patients with schizophrenia.

Smrt *et al* demonstrated that miR-137 serves as a regulator of adult neural stem cell maturation and migration to the subventricular zone, located around the lateral ventricles, and the subgranular zone of the hippocampus (35). In addition, miR-137 was shown to be highly associated (P=1.6×10^−11^) with schizophrenia in one of the largest genome-wide association studies (36). Since miRNAs are crucial regulators of gene expression, important genetic mechanisms may contribute to the phenotypic heterogeneity. Lett *et al* made demographics for age-at-onset samples, as well as healthy controls, and their findings indicated that miR-137 plays a considerable role in the variation in phenotypes that is believed to have an important role in clinical outcome and treatment response (20). The authors concluded that the effects of miR-137 on the phenotypic heterogeneity of schizophrenia may occur via neurodevelopmental gene networks. These observations may provide a model for the role of miRNAs in the phenotypic heterogeneity of psychiatric disorders.

## 3. miRNAs may be potential biomarkers in the diagnosis of schizophrenia

The diagnosis of schizophrenia is currently based exclusively on signs and symptoms; therefore, a diagnosis requires qualified psychiatric assessment. The majority of studies investigating the genetics of schizophrenia have been limited to protein-coding genes. However, the regulatory role of miRNAs has received increasing attention, with results indicating that miRNAs may contribute to the etiology of schizophrenia. miRNAs are known to influence complex gene networks and pathways, which suggests that they may be potential biomarkers when dysregulated. miRNAs as biomarkers have been shown to be useful in the clinical stratification of neoplasms, and even have a greater prognostic significance compared with mRNAs, which may result from miRNAs being discrete functional entities (36). The expression of peripheral miRNA has also been examined in other neurological disorders. For instance, in Alzheimer’s disease, miRNA was found to be altered in peripheral blood mononuclear cells (PBMCs) (37), cerebrospinal fluid and brain tissue (38).

Gardiner *et al* investigated the expression profile of miRNA in PBMCs of 112 patients with schizophrenia and 76 non-psychiatric controls (21). The authors identified 83 miRNAs that were significantly downregulated in the schizoaffective group, including a large subgroup of miRNAs (20%) transcribed from a single imprinted locus at the maternally expressed DLK1–DIO3 region on chromosome 14q32. Similarly, Lai *et al* identified a signature of seven miRNAs in an initial cohort of 30 patients with schizophrenia and 30 controls, which included the upregulated miR-34a, miR-449a, miR-564, miR-548d, miR-572 and miR-652, and downregulated miR-432 (22). The results of the study were subsequently validated in an extended cohort of 60 schizophrenia patients and 30 controls. The expression levels of a number of these miRNAs were found to be correlated with negative symptoms, neurocognitive dysfunction and mismatched negativity performances of the schizoaffective patients. Notably, miR-449a was shown to be closely associated with the majority of features examined in the Wisconsin Card Sorting Test (39), indicating the possible involvement of miR-449a in the executive function of the brain (36).

The majority of previous blood-based gene expression studies on schizophrenia have been limited to the expression of protein-coding genes. A large number of mRNAs have been detected using microarray platforms (40–43), leading to a number of putative mRNAs being associated with schizophrenia; however, the majority of associations have not been possible to replicate in other studies (44). The regulatory role of miRNAs has received increasing attention since miRNAs regulate the expression levels of genes by inhibiting the translation of mRNAs and may be potential biomarkers for schizophrenia (45–49). Since each miRNA can regulate the expression levels of hundreds of target genes, the number of discriminating miRNAs as biomarkers is likely to be much less compared with mRNAs.

Therefore, the aforementioned studies have demonstrated that the peripheral patterns of miRNAs may be used as biomarkers for schizophrenia and are associated with subphenotypes.

## 4. Summary and future prospects

Schizophrenia exhibits a complex neurobehavioral phenotype that is considered to have developed through turbulences in the neural circuitry and synaptic function. Due to the abundance of neural networks, various combinations of gene variants can cause system dysfunctions, manifesting as associated neurobehavioral syndromes. miRNAs appear to be important in neural networks and are highly expressed in the brain, emerging as key regulators of numerous neurodevelopmental and neurological processes. Dysregulation of miRNAs leads to pervasive changes and defects of the nervous system, which may assist investigations into the pathophysiology and neuropathology of schizophrenia. Due to their tuning effect on numerous proteins, miRNA biomarkers for schizophrenia and associated phenotypes may ultimately provide the basis for early detection, disease stratification and prediction of response to drugs and side-effects; however, mood swings can affect the expression of certain miRNAs (50), and this may be a disadvantage of using miRNAs to diagnose schizophrenia.

miRNAs have the following advantages: i) miRNA remains stable despite changes in temperature, pH or physical state; ii) the expression of miRNAs is specific with no difference in gender or in individual; iii) miRNAs in serum and plasma can be quantified *in vitro* (51); thus, miRNAs are promising biomarkers instead of proteins. To date, miRNAs extracted from brain tissue, cerebrospinal fluid and PBMCs have been used as biomarkers in the diagnosis of schizophrenia; furthermore, the decoding of the miRNA genome may contribute to elucidating the etiology and improving the treatment of schizophrenia. miRNAs extracted from other peripheral sources, however, have not been investigated. Future studies should investigate miRNAs from other peripheral fluids, including saliva and urine, as these may also be potential biomarkers in the diagnosis of schizophrenia.

## Figures and Tables

**Table I:** Schizophrenia-associated miRNA. Adapted from the study by Beveridge and Cairns (36).

Study	Type	Brain region	Schizophrenia-associated miRNA
Lett *et al*, 2013 (20)	GWAS	Hippocampi, white matter	miR-137
Gardiner *et al*, 2011 (21)	Peripheral tissue (PBMC)	N/A	[Downregulated]miR-107,miR-1275,miR-128,miR-130b^*^,miR-134,miR-148b, miR-150^*^, miR-151-3p, miR-16-2^*^, miR-181a, miR-200c, miR-224, miR-28-3p, miR-28-5p, miR-29b-1^*^, miR-30e^*^, miR-31, miR-329, miR-335^*^, miR-342-5p,miR-409-3p,miR-431,miR-432,miR-486-3p,miR-487b,miR-544, miR-574-3p, miR-576-5p, miR-584, miR-625^*^, miR-664, miR-877, miR-99b
Lai *et al*, 2011 (22)	Peripheral tissue (PBMC)	N/A	[Upregulated] miR-34a, miR-449a, miR-548d, miR-564, miR-572, miR-652 [Downregulated] miR-432
Moreau *et al*, 2011 (23)	Postmortem brain	DLPFC (BA9)	[Upregulated] miR-148b, miR-151 miR-27b, miR-301, miR-545, miR-639 [Downregulated] miR-106b, miR-138, miR-193b, miR-210, miR-22, miR-324-3p, miR-338, miR-339, miR-425
Santarelli *et al*, 2011 (24)	Postmortem brain	DLPFC (BA9)	[Upregulated] miR-105, miR-134, miR-148b, miR-150, miR-152, miR-154, miR-17-5p, miR-187, miR-193a, miR-199a^*^, miR-199b, miR-222, miR-25, miR-328, miR-382, miR-409-3p, miR-423, miR-425-5p, miR-433, miR-452^*^, miR-487a, miR-495, miR-502, miR-512-3p, miR-519c, miR-532, miR-542-3p, miR-548b, miR-590, miR-592, miR-652, miR-767-5p, miR-92b
The Schizophrenia Psychiatric GWAS Consortium, 2011	GWAS	N/A	miR-137
Beveridge *et al*, 2010 (25)	Postmortem brain	STG (BA22)	[Upregulated] let-7e, miR-107, miR-125b, miR-128a, miR-128b, miR-129, miR-130a, miR-133b, miR-138, miR-146b, miR-148a, miR-150, miR-152, miR-155, miR-15a, miR-15b, miR-16, miR-17-3p, miR-17-5p, miR-181b, miR-195, miR-197, miR-199a^*^, miR-19a, miR-20a, miR-222, miR-23a, miR-24, miR-26b, miR-26b, miR-27b, miR-28, miR-296, miR-328, miR-330, miR-335, miR-338, miR-339, miR-340, miR-373^*^, miR-381, miR-409-5p, miR-432^*^, miR-452^*^, miR-455, miR-484, miR-485-5p, miR-486, miR-487a, miR-489, miR-494, miR-499, miR-502, miR-517a, miR-517c, miR-518b, miR-519d, miR-520a^*^, miR-520g, miR-9^*^, miR-99a
Beveridge *et al*, 2010 (25)	Postmortem brain	DLPFC (BA9)	[Upregulated] let-7d, miR-101, miR-105, miR-107, miR-126^*^, miR-128a, miR-153, miR-15a, miR-15b, miR-16, miR-16, miR-181a, miR-181b, miR-181b, miR-181d, miR-184, miR-195, miR-199a, miR-20a, miR-219, miR-223, miR-26b, miR-27a, miR-29c, miR-302a^*^, miR-302b^*^, miR-31, miR-33, miR-338, miR-409-3p, miR-512-3p, miR-519b, miR-7
Kim *et al*, 2010 (26)	Postmortem brain	DLPFC (BA46)	[Upregulated] miR-132, miR-132^*^, miR-154^*^, miR-212, miR-34a, miR-544, miR-7
Mellios *et al*, 2010 (27)	Postmortem brain	Frontal cortex (BA10)	[Downregulated] miR-30b
Xu *et al*, 2010 (28)	miR-SNP	N/A	miR-24, pre-miR-30e, miR-30e
Feng *et al*, 2009 (29)	miR-SNP	N/A	let-7f-2, miR-188-3p, pre-miR-18b, miR-325-3p, pre-miR-502, pre-miR-505, miR-509-3p, miR-510-3p, miR-660
Mellios *et al*, 2009 (30)	Postmortem brain	Frontal cortex (BA10)	[Downregulated] miR-30e, miR-195
Sun *et al*, 2009 (31)	miR-SNP	N/A	miR-502, miR-510
Tabares-Seisdedos *et al*, 2009 (32)	CNV	N/A	miR-124-1, miR-320, miR-383, miR-486, miR-596, miR-597, miR-598
Zhu *et al*, 2009 (3)	Postmortem brain		DLPFC (BA46) miR-346
Beveridge *et al*, 2008 (33)	Postmortem brain		STG (BA22) miR-181b
Hansen *et al*, 2007 (34)	miR-SNP	N/A	miR-198, miR-206
Perkins *et al*, 2007 (18)	Postmortem brain	DLPFC (BA9)	[Upregulated] miR-106b, miR-7 [Downregulated] miR-26b, miR-30b, miR-29b, miR-195, miR-92, miR-30a, miR-30d, miR-20b, miR-29c, miR-29a, miR-212, miR-24, miR-30e, miR-9^*^

These miRNAs have been shown to exhibit altered expression levels in numerous studies and have exhibited consistent changes in schizophrenia. miRNA, microRNA; GWAS, genome-wide association study; PBMC, peripheral blood mononuclear cells; DLPFC, dorsolateral prefrontal cortex; BA, Brodmann’s area; miR-SNP, single nucleotide polymorphism within a microRNA; STG, superior temporal gyrus; CNV, copy-number variant.
